# Influence of Urban and Rural Areas, Type of School, and Parents’ Education Level on Nutrition Habits and Their Relationship with Dental Caries in Schoolchildren in Mallorca

**DOI:** 10.3390/children12030383

**Published:** 2025-03-19

**Authors:** Daniela Vallejos, Irene Coll, Nora López-Safont

**Affiliations:** 1 Facultad of Dentistry, University ADEMA School, 07009 Palma, Spain; d.vallejos@eua.edu.es (D.V.); i.coll@eua.edu.es (I.C.); 2 Health Group of University Institute for Research in Health Sciences (IUNICS), 07122 Palma, Spain; 3 Biology Department, University of Balearic Islands, Ctra. Valldemossa Km 7.5, 07122 Palma, Spain

**Keywords:** dental caries, nutrition, sociodemographic factors

## Abstract

**Background/Objectives**: Habits such as a diet high in sugars and poor dental biofilm control are linked to a higher prevalence of caries and low socioeconomic status. This study aimed to analyze the nutrition habits of schoolchildren in Mallorca and their relationship with the presence of dental caries, depending on the type of school, geographic location, and parents’ education level. **Methods:** A cross-sectional study was conducted to examine the prevalence of dental caries based on World Health Organization (WHO) standards and nutritional practices following guidelines from the Food and Agriculture Organization of the United Nations (FAO) and the European Food Safety Authority (EFSA). This study included 718 students from three age groups: first-year elementary students (ages 5–6), sixth-year elementary students (age 12), and fourth-year secondary school students (age 15). Relevant sociodemographic factors were also considered in the analysis. **Results:** In schoolchildren aged 5–6 years, higher monthly consumption of processed and sugary foods, such as sweets (rural: 24.66 (CI 95%: 20.30–29.02); urban: 19.29 (CI 95%:16.27–22.304); *p* = 0.044), was noted in schoolchildren from rural sectors compared to those residing in urban areas. At 15 years of age, there was a higher consumption of potato chips in public schools than in subsidized/private schools (public: 26.95 (CI 95%: 24.42–29.49); subsidized/private: 18.29 (CI 95%: 13.92–22.65) *p* = 0.004). A high consumption of sweets is associated with an increased risk of caries (OR sweets: 1.76 CI: 1.04–2.98; *p* = 0.035). Fewer students with mothers with a lower education level eat dinner (elementary: 75%; secondary 91%; higher: 98%; *p* = 0.003). **Conclusions:** Higher consumption of sweets in rural areas and potato chips in public schools, along with the association between sweet consumption and caries risk, highlight how geographic location, school type, and parents’ education level influence children’s nutrition habits and caries.

## 1. Introduction

The developmental years between childhood and adolescence are a fundamental period of socialization, during which eating habits are formed that are maintained into adulthood. This process is affected by socioeconomic, cultural, and environmental factors [1]. Thus, childhood experience is crucial to future health due to the continuous malleability of biological systems, and there is evidence that healthy habits, such as good nutrition and exercise, are influenced by the examples of parents and peers [2]. Students spend a significant part of their day at school, so it is reasonable to expect that the school environment plays an important role in teaching and shaping eating habits [3].

Dietary patterns have important implications, especially for the prevention or development of chronic diseases. Although children’s eating habits are difficult to change directly, parental eating habits are considered a good target for interventions aimed at preventing unhealthy eating behaviors in children [4,5].

Dental caries is a non-communicable disease strongly linked to lifestyles and harmful behaviors, mainly related to inadequate dietary patterns, and is linked to a dysbiosis of the dental biofilm caused by exposure to free sugars [6].

The most relevant modifiable behavioral factors correlating with a higher prevalence of caries are a diet high in sugars and poor dental biofilm control. Many studies seem to indicate that these unhealthy habits are associated with low income [6,7,8,9,10].

The frequency and type of foods consumed as well as dietary consistency significantly impact oral health. Diets high in sugars and carbohydrates are associated with an increased risk of dental caries, periodontal disease, and oral cancer. In addition, oral health influences the ability to maintain a nutritious diet, affecting chewing, taste perception, and nutritional intake [11].

Additional research indicates that processed food containing sugar and starch increases the risk of dental caries [12]. In contrast, the intake of natural sugars from cereals, fruits, vegetables, and milk does not substantially contribute to the onset of dental caries or other non-communicable diseases, owing to protective elements such as fiber, water content, polyphenols, and calcium [11,12]. In addition, chewing these foods stimulates salivary flow, mitigating the risk from sugars [13,14].

The amount and frequency of sugar consumption are risk factors for developing dental caries. Animal and human studies have shown that both variables significantly influence the development of caries [14,15,16]. Therefore, both the amount and frequency of sugar intake should be considered to prevent caries and other non-communicable diseases. However, the relationship between other nutritional patterns, such as skipping meals or eating outside the home, has not been as extensively studied.

Despite the increasing body of research regarding the link between nutrition and oral health, no studies have specifically investigated this relationship in schoolchildren in Mallorca.

This study aimed to analyze the influence of the type of school, geographic location, and parents’ education level on the nutrition habits of schoolchildren in Mallorca and their relationship with the presence of dental caries.

## 2. Materials and Methods

### 2.1. Study Design and Target Population

A cross-sectional observational epidemiological study was conducted in the school population in Mallorca between October 2018 and December 2019. The sample size was calculated as follows. For a population of 12,000 children and a caries prevalence of 0.35 extracted from the National Institute of Statistics, the minimum sample size was approximately 340 children to achieve a 95% confidence level with a 5% margin of error. However, we wanted to increase the sample size to enhance the accuracy of our effect estimates, reduce the margin of error, and increase the statistical power of this study. For that, we analyzed 718 students in three age groups—5–6 years (first-year elementary school), 12 years (sixth-year elementary school), and 15 years (fourth-year secondary school)—in the 28 schools selected. The epidemiological study design used a stratified cluster sampling technique, following the Pathfinder method [17]. This method allows the most important population subgroups that may have different levels of disease to be included. The selected age groups are WHO-designated “index ages”, recommended for examination. Schools were chosen using a systematic random sampling method, grouping the sample into three strata based on the population characteristics of Mallorca, as shown in Table 1.

The sampling sites (schools) for the study population were identified using data from the General Directorate of Planning, Organization, and Schools of the Autonomous Community of the Balearic Islands (CAIB) and the National Institute of Statistics (INE). Students aged 5–6, 12, and 15 years were selected, corresponding to first-grade and sixth-grade elementary and fourth-year secondary school in the chosen schools across Mallorca. The design, protocol, and methodology of the transversal study are described in more detail elsewhere [17,18].

Fieldwork and data collection were conducted on-site at the schools. Seven examining dentists performed the inspections under standardized conditions, utilizing a headlamp, a #5 intraoral flat mirror, and a WHO periodontal probe according to the WHO recommendations for oral health surveys [19]. The calibration and training of the examining dentists followed WHO guidelines. The simple agreement rate was 98.7%, and the Kappa index, evaluated using the Landis and Koch scale, was 0.757. This indicated a high level of agreement (greater than 95% for simple agreement and greater than 0.61 for Kappa), which was deemed sufficient to commence this study.

The data were collected using the form specified by the WHO Oral Health Surveys: Basic Methods [19] (Annex 2 of the Oral Health Surveys: Basic Methods Manual) and the oral health questionnaire for children (Annex 8 of the same manual).

The nutrition habits of students were also studied through surveys that provide information on diet-related knowledge, attitudes, and practices, adaptable KAP questionnaires extracted from the FAO publication “Guidelines for assessing nutrition-related Knowledge, Attitudes and Practices” (FAO, 2014) [20]. A questionnaire on food consumption patterns was also administered, following the guidelines proposed by the European Food Safety Authority (EFSA, European Food Safety Authority). These guidelines are part of the “EUMenu Project” in Europe and are included in the European methodological guide of 2009, “General principles for the collection of national food consumption data in the view of a pan-European dietary survey” (EFSA, 2009) [21].

The questionnaires on knowledge and nutritional habits were administered by a multidisciplinary team of health care professionals (dentists, doctors, biologists, and nutritionists) who posed the questions to the schoolchildren and recorded the responses. Unanswered questions were considered missing values (excluded from the analysis). The design, protocol, and methodology of the surveys are described elsewhere [22].

### 2.2. Study Variables

The study variables are as follows.

Sociodemographic variables:

- Age;
- Type of school: public or subsidized/private;
- Geographic location: urban or rural;
- Education level of parents/guardians: includes the educational level of both the mother and father, categorized as elementary, secondary, or higher education.

Nutritional variables:

- Frequency of food consumption: the number of times consumed per month;
- Nutrition-related attitudes and practices: an assessment of the number of meals per day, where they were eaten, and the purchase of snacks.

Oral health variables:

- Experience of caries is determined through the DMFT index in permanent teeth, where D=decayed teeth, M=missing teeth due to caries, and F=filled teeth corresponding to the unit being assessed. The analysis of caries experience uses the total sample (for children aged 5–6 years, among those who had mixed dentition, only permanent teeth were considered).

### 2.3. Ethical Treatment of the Data

Prior to beginning this study, approval was granted from the ethics board of the Balearic Islands (CEI: IB3737/18) in compliance with prevailing legislation, and this study was implemented in conformity with the principles outlined in the Declaration of Helsinki and the standards of good clinical practice. Before data collection, the data sheet and the informed consent form were given to the children’s parents/guardians; only those children who had previously submitted the consent form signed and dated by their parents/guardians participated in this study.

All the collected data were entered into an automated file with restricted access, and personal data were encrypted to guarantee confidentiality.

### 2.4. Statistical Analysis

The data were analyzed using the SPSS 27.0.1.0^®^ statistics application, depending on the type of variable and the groups to be analyzed. The effects of the following factors on the different variables were assessed: the type of school (public or subsidized/private), geographic location (urban or rural), and education level of the mothers/guardians and fathers/guardians (elementary, secondary, and higher education). To compare means, Student’s *t*-test or a one-way analysis of variance (ANOVA) was used in conjunction with the Bonferroni post hoc analysis. The percentages were compared with chi-square tests using the cross-table procedure—the cross-tabulation tool analyzed risk by calculating the odds ratio (OR), and this analysis was used to determine the effect size. In every case, the 95% confidence interval estimate (*p* < 0.05) was used to determine the precision of the random error present in the data.

## 3. Results

### 3.1. Sample Description

During the period in which this study was conducted, 718 students in the first year of elementary school (5–6 years), sixth year of elementary school (12 years), and fourth year of secondary school (15 years) were explored. Table 2 shows the students’ distribution according to age, sex, and school type.

### 3.2. Nutrition Habits

#### 3.2.1. Frequency of Food Consumption

From the data collected through the survey on the frequency of consumption of the food groups that form part of the students’ diets, foods that are related to the development of dental caries were selected and detailed by the children’s age.

In the group of 5–6 year olds, higher consumption of processed and sugary foods such as pastries, sausages, sugary soft drinks, commercial juices, and sweets in children from rural sectors than those residing in urban areas is illustrated in Table 3.

In the group of 12 year olds, the results show a higher frequency of consumption of foods such as pastries, potato chips, sugary soft drinks, and sweets in rural students compared to those living in urban areas. No differences were observed according to the type of school attended, as shown in Table 4.

In the case of the 15 year olds, higher consumption of potato chips and sausages was observed in students from rural areas and public schools compared to urban areas and subsidized or private schools. Commercial juices were consumed more frequently in public schools, as shown in Table 5.

**Figure 1:**
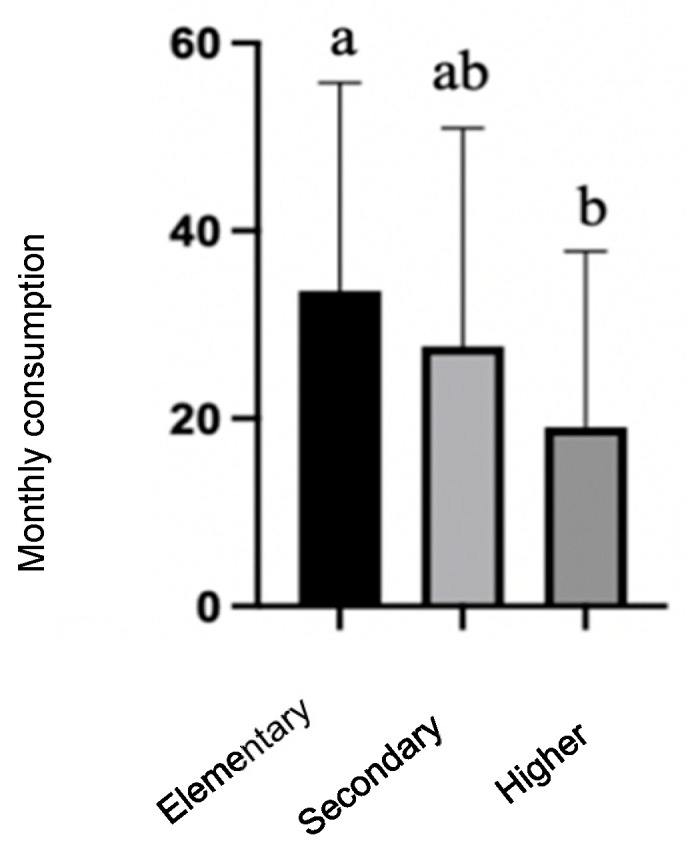
Frequency of monthly sausage consumption in 15 year olds according to the parents/guardian’s education level. Columns that do not share the same letter (a, b) have significant differences (ANOVA, *p* < 0.05, and Bonferroni post hoc analysis).

The results indicate that a lower education level of the parents/guardians was associated with a higher consumption of sausages. The mean frequency of consumption was significantly higher in students with parents who had only elementary education (33.57 ± 22.190) than in those with parents/guardians with higher education (19.08 ± 18.72; *p* = 0.004), as shown in Figure 1.

#### 3.2.2. Meals During the Day

Another important factor that was evaluated to assess nutrition habits was whether schoolchildren consumed all the main meals of the day and where they ate them.

Snacks between meals and whether they bought these foods with their own money were also considered.

As illustrated in Table 6, in the 5–6-year-old group, more children from urban areas (99.3%) ate breakfast at home than schoolchildren from rural areas (93.7%; *p* = 0.043).

Table 7 shows that a higher proportion of 12 year olds in urban areas (97.1%) ate lunch than those in rural areas (86.3%; *p* = 0.010). Additionally, 5–6 year olds from rural areas (90.1%) and public schools (85.9%; *p* = 0.037) tended to have lunch at home in a higher proportion than students from urban areas (77.6%) and subsidized/private schools (73.6%; *p* = 0.043).

Table 8 shows the proportion of students who ate dinner and where they ate it; in this case, no significant differences were observed between the groups.

Among 15 year olds, a higher percentage of students in rural areas (29%) and public schools (25.5%; *p* = 0.004) reported purchasing snacks with their own money compared to those in urban areas (13%) and subsidized/private schools (7%; *p* = 0.008).

#### 3.2.3. Parents/Guardians’ Education Levels

The findings indicate that the education level of the mother/guardian affected the proportion of 15 year olds who had dinner. Fewer adolescents who ate dinner were noted among students with mothers with a lower education level (elementary: 75%; secondary 91%; higher: 98%; *p* = 0.003).

The influence of the father/guardian’s education level was related to where the 15 year olds had dinner; a higher education level was related to a higher proportion of children eating at home (elementary: 93%; secondary 98%; higher 100%; *p* = 0.031).

### 3.3. Effect of Nutrition Habits on the Experience of Caries in Schoolchildren

#### 3.3.1. Consumption of Sugary Foods

In an analysis of the impact of food consumption on students’ experience of caries, using an OR analysis, we found an increased likelihood of caries in permanent teeth among schoolchildren who reported a higher frequency of sweets consumption than those with lower consumption rates (OR= 1.757; CI= 1.036–2.978; *p* = 0.035), as shown in Table 9. According to our results, the other processed foods were unrelated to a higher caries frequency.

#### 3.3.2. Meals During the Day

The results show that habits that respected compliance with the main meals were related to better oral health. As shown in Table 10, a higher proportion of students with experience of caries ate breakfast (93.6% vs. 86.7; *p* = 0.004) and dinner (97.6% vs. 92.8%; *p* = 0.004) than those with an experience of caries.

Conversely, where lunch was eaten seemed to influence the experience of caries. More children with an experience of caries ate lunch outside of home or school (19%) than children with no experience of caries (11% *p* = 0.009).

The results show no significant difference in the experience of caries between schoolchildren who ate snacks (yes: 64.4%; no: 35.6% *p* = 0.853) and those who bought food with their own money (yes: 18.9%; no: 81.1% *p* = 0.310)

## 4. Discussion

The data analysis in this study indicates that the sociodemographic factors analyzed, such as geographic location, school type, and parents’ level of education, influenced the nutrition habits of the schoolchildren who participated in the project. In addition, we noted that some of these habits were related to the experience of caries in students.

The mechanisms through which the environment affects the health behaviors of children and adolescents have been articulated in multiple ways, with some authors indicating that the social groups in which children engage transmit their social norms and attitudes by filtering and adapting the media messages that children encounter [4]. On the other hand, it is considered that the perception and knowledge shared about a healthy lifestyle are influenced by factors such as communication, socioeconomic level, and parenting practices and style [23].

Our findings suggest that the parents’ education level influences nutrition habits; a higher education level correlates with a lower consumption of some processed foods, such as sausages, and a greater number of children who eat dinner at home. Evidence has shown that children’s food preferences are related to parental eating habits, economic conditions, and nutritional knowledge [24]. The mother’s education level significantly affects the dietary habits of children and adolescents; children of mothers with low education levels exhibit increased consumption of sugar, fat, and protein [4,25].

Although we did not directly determine the socioeconomic levels of the students who participated in this study, the data provided by the National Statistics Institute (INE) [26] show that private or subsidized schools are always concentrated in high-income areas, unlike public schools, most of which are in areas with fewer resources, with a predominance of low and lower-middle-class students [27]. Regarding geographic location, our data showed that the proportion of caregivers with higher education is greater in urban than rural areas. These differences have been attributed to the greater difficulty that rural inhabitants have in accessing health care, education, job opportunities, technology, public transportation, and commerce [28,29]. This means that rural regions have distinct economic, social, and structural characteristics that lead to the development of social disparities and exacerbate social inequalities in health.

There is evidence of poorer oral health in rural areas, and this is associated with factors such as limited access to oral health care, low education levels, and less healthy lifestyles [8,9,10]. For example, adults living in rural areas visit the dentist less often, have less frequent cleanings, and have more extractions of permanent teeth than adults in urban areas [30].

These factors also exerted a certain influence on the students’ dietary habits in this study, and this is reflected in our results, where we noted more frequent consumption of some processed and sugary foods such as pastries, sugary soft drinks, commercial juices, sausages, potato chips, and sweets in rural areas and public schools compared to subsidized/private schools located in urban areas. We observed that schoolchildren who reported a higher frequency of candy consumption had a higher probability of caries in their permanent teeth, while sugary drinks and sodas did not show the same association. The possible reasons why only candy consumption was statistically significant may be related to consumption patterns. Sweets are generally eaten between meals, increasing prolonged exposure to sugar, while sugary drinks and sodas are typically consumed with main meals. These observations have a certain level of limitation in the 5–6-year-old group, as primary teeth were not considered in the analysis and may not fully reflect the impact of nutritional habits on caries in this age group; this limitation can be addressed in future studies.

In addition, there was greater compliance with the three daily meals in urban areas than in rural areas. These findings are relevant if we consider that some authors point out that dietary quality is influenced by practices such as breakfast [31,32] and that children who eat breakfast at home with their parents have a lower consumption of sugary drinks [33].

These disparities are ascribed to many variables; some authors characterize those with limited means as being at an elevated risk of experiencing economic hardship, rendering them more susceptible to consuming a less nutritious diet. This is because low-cost and more energetic foods are readily available in socioeconomically deprived areas, in addition to the influence of a higher education level on healthier nutrition habits related to knowledge, beliefs, and the ability to assimilate nutritional information [34,35,36,37].

Conversely, our study revealed an increased likelihood of caries in permanent teeth among schoolchildren who reported a higher frequency of sweet consumption, aligning with the existing literature that indicates a heightened risk of caries in individuals who frequently eat sugary foods [6].

Our findings indicate that habits such as eating breakfast and dinner correlate with a reduced incidence of caries. Additionally, where lunch is consumed appears to impact the oral health of schoolchildren, which can be explained by the association of these dietary patterns with a healthier diet [31,32].

The findings from the 2020 Spanish Oral Health Survey emphasize the ongoing challenges related to socioeconomic disparities in oral health, as children from lower socioeconomic backgrounds tend to have higher caries rates [38]. The survey shows improvements in dental health across various age groups. For 5–6 year olds, the DMFT index for permanent teeth is 0.06; in 12 year olds, the DMFT index for permanent teeth is 2.03, and in 15 year olds, it is 2.66 [38]. In fact, we can observe the same situation in the Balearic Islands in a previous study. Regarding the prevalence of caries prevalence, we indicate that at the ages of 5–6, 12, and 15, the trend is the same, dropping from 45.5% to 40%, from 34.9% to 27.9%, and from 60.2% to 45.49%, respectively (in 10 years) [18].

Although this study focused on nutritional habits, it would be interesting to explore in future studies the relationship between sociodemographic variables, such as gender, education level, geographic location, type of school, and parental education, and the prevalence of dental caries in children from Mallorca. This approach would provide a more comprehensive understanding of the factors influencing children’s dental health.

A limitation of this study is the sample size, which is focused only on the Mallorca population. Expanding the sample to include a broader, more diverse group from different regions of Spain or the Balearic Islands would enhance the generalizability of the findings. Additionally, due to the cross-sectional design, the main weakness of this study is the lack of follow up, which limits the ability to establish a causal relationship.

## 5. Conclusions

According to the results, the type of school, geographic location, and parents’ education level seem to influence children’s eating habits. The dietary habits of schoolchildren, particularly the consumption of sweets, were associated with the prevalence of caries. While the evidence suggests a link between dietary habits and oral health, further investigation is needed to fully understand the broader impact on overall health and the role of the surrounding environment in shaping these habits.

## Figures and Tables

**Table 1:** Description of the different strata comprising the sample.

First Stratum	Second Stratum	Third Stratum
Population Nuclei:	School Types:	Age Groups:
- Urban/Peri-urban - Rural	- Public - Subsidized/Private	- 5–6 years (first-grade elementary) - 12 years (sixth-grade elementary) - 15 years (fourth-year secondary)

Stratified cluster sampling technique.

**Table 2:** Distribution of the sample by grade, sex, and school type.

		First-Year Elementary (5–6 Years Old) n = 255		Sixth-Year Elementary (12 Years Old) n = 230		Fourth-Year Secondary (15 Years Old) n = 233		Total	
		n	%	n	%	n	%	n	%
Sex	Male	144	56.47	125	54.35	112	48.06	381	53.06
	Female	111	43.53	105	46.65	121	51.93	337	46.94
School type	Public	177	69.4	159	69.1	190	81.5	526	73.3
	Subsidized/private	78	30.6	71	30.9	43	18.5	192	26.7
Geographic location	Urban	163	63.9	140	60.9	101	43.3	404	56.3
	Rural	92	36.1	90	39.1	132	56.7	314	43.7

n, sample size.

**Table 3:** Mean frequency of food consumption per month according to geographic location and school type: 5–6 year olds.

	Geographic Location	n	Mean	SD	CI 95%	*p*-Value	School Type	n	Mean	SD	CI 95%	*p*-Value
Pastries	Rural	85	27.62	21.94	(22.96–32.29)	0.013 *	Public	169	23.88	21.73	(20.61–27.16)	0.307
	Urban	160	20.45	20.93	(17.20–23.69)		Subs./priv.	76	20.84	21.02	(16.12–25.57)	
Unknown		10						10				
Potato chips	Rural	85	19.84	15.82	(16.47–23.20)	0.192	Public	169	18.82	15.91	(16.42–21.21)	0.243
	Urban	160	17.06	15.79	(14.62–19.51)		Subs./priv.	76	16.26	15.59	(12.76–19.77)	
Unknown		10						10				
Sausages	Rural	81	30.54	23.34	(25.46–35.63)	<0.001 *	Public	162	24.79	22.77	(21.28–28.30)	0.071
	Urban	155	19.12	20.39	(15.90–22.32)		Subs./priv.	74	19.19	20.11	(14.61–23.77)	
Unknown		19						19				
Sugar	Rural	82	22.34	22.71	(17.43–27.26)	0.699	Public	160	24.23	23.94	(20.52–27.94)	0.290
	Urban	149	23.58	23.65	(19.79–27.38)		Subs./priv.	71	20.70	21.69	(15.66–25.75)	
Unknown		24						24				
Sugary soft drinks	Rural	86	41.58	22.73	(36.78–46.38)	<0.001 *	Public	168	36.48	24.58	(32.76–40.19)	0.002
	Urban	156	28.56	25.25	(24.60–32.53)		Subs./priv.	74	25.73	24.90	(20.06–31.40)	
Unknown		13						13				
Commercial juices	Rural	87	24.07	23.52	(19.13–29.01)	0.005 *	Public	167	20.10	21.46	(16.84–23.35)	0.268
	Urban	152	16.30	18.00	(13.44–19.16)		Subs./priv.	72	16.89	17.95	(22.74–31.04)	
Unknown		16						16				
Sweets	Rural	85	24.66	20.52	(20.30–29.02)	0.044 *	Public	168	22.68	20.82	(19.53–25.83)	0.076
	Urban	157	19.29	19.30	(16.27–22.30)		Subs./priv.	74	17.76	17.124	(14.54–20.97)	
Unknown		13						13				
Fruits	Rural	85	9.88	15.80	(6.52–13.24)	0.004 *	Public	167	13.21	18.14	(10.45–15.96)	0.158
	Urban	157	16.68	18.32	(13.81–19.54		Subs./priv.	75	16.69	16.72	(12.91–20.48)	
Unknown		13						13				
Vegetables	Rural	86	14.41	18.30	(10.55–18.29)	0.176	Public	167	16.40	18.97	(13.52–19.27)	0.799
	Urban	157	17.80	18.68	(14.87–20.72)		Subs./priv.	76	17.05	17.81	(13.05–21.06	
Unknown		13						12				

n, sample size; SD, standard deviation; Student’s *t*-test *p*-values; * variable with significant effect *p* < 0.05.

**Table 4:** Mean frequency of food consumption per month according to geographic location and school type: 12 year olds.

	Geographic Location	n	Mean	SD	CI 95%	*p*-Value	Type of School	n	Mean	SD	CI 95%	*p*-Value
Pastries	Rural	88	29.32	20.21	(25.10–33.54)	0.008 *	Public	157	22.56	19.13	(19.57–25.55)	0.017
	Urban	136	21.76	20.98	(18.24–25.29)		Subs./priv.	67	29.82	24.13	(24.04–35.60)	
Unknown		6						6				
Potato chips	Rural	88	26.48	16.52	(23.03–29.93)	<0.001 *	Public	156	21.90	15.96	(19.39–24.40)	0.985
	Urban	136	18.91	16.23	(16.18–21.64)		Subs./priv.	68	21.85	18.48	(17.46–26.24)	
Unknown		6						6				
Sausages	Rural	86	25.05	21.72	(20.46–29.64)	0.236	Public	155	22.66	20.54	(19.42–25.89)	0.740
	Urban	136	21.65	20.16	(18.26–25.04)		Subs./priv.	67	23.67	21.50	(18.52–28.82)	
Unknown		8						8				
Sugar	Rural	86	19.93	20.01	(15.70–24.16)	0.688	Public	154	19.74	20.13	(16.56–22.92)	0.329
	Urban	133	21.10	21.58	(17.43–24.77)		Subs./priv.	65	22.77	22.76	(17.24–28.30)	
Unknown		8						11				
Sugary soft drinks	Rural	88	32.43	20.72	(28.10–36.76)	<0.001 *	Public	155	25.69	22.03	(22.22–29.16)	0.743
	Urban	134	21.79	21.72	(18.11–25.47)		Subs./priv.	67	26.74	21.77	(21.53–31.96)	
Unknown		8						8				
Commercial juices	Rural	84	24.67	21.40	(20.09–29.24)	0.443	Public	150	22.16	22.28	(18.60–25.73)	0.336
	Urban	134	22.22	23.72	(18.21–26.24)		Subs./priv.	68	25.38	24.05	(19.67–31.10)	
Unknown		8						12				
Sweets	Rural	87	24.48	15.24	(21.28–27.69)	0.024 *	Public	155	20.67	15.91	(18.17–23.18)	0.426
	Urban	136	19.22	17.84	(16.22–22.22)		Subs./priv.	68	22.65	19.42	(18.03–27.26)	
Unknown		7						7				
Fruits	Rural	87	9.95	12.99	(7.23–12.68)	0.120	Public	156	12.71	15.73	(10.24–15.17)	0.269
	Urban	137	13.23	16.59	(10.45–16.00)		Subs./priv.	68	10.24	14.37	(6.82–13.65)	
Unknown		6						7				
Vegetables	Rural	87	16.18	17.67	(12.47–19.90)	0.485	Public	152	14.61	16.26	(12.02–17.19)	0.413
	Urban	133	14.59	15.81	(11.90–17.27)		Subs./priv.	68	16.59	17.22	(12.50–20.68)	
Unknown		10						10				

n, sample size; SD, standard deviation; Student’s *t*-test *p*-values; * variable with significant effect *p* < 0.05.

**Table 5:** Mean frequency of consumption per month according to geographic location and school type: 15 year olds.

	Geographic Location	n	Mean	SD	CI 95%	*p*-Value	School Type	n	Mean	SD	CI 95%	*p*-Value
Pastries	Rural	130	22.54	19.13	(19.25–25.83)	0.854	Public	188	23.30	18.79	(20.61–25.98)	0.335
	Urban	101	22.99	17.71	(19.54–26.45)		Subs./priv.	43	20.28	17.12	(15.16–25.40)	
Unknown		2						2				
Potato chips	Rural	131	28.18	18.28	(25.05–31.31)	0.005 *	Public	189	26.95	17.77	(24.42–29.49)	0.004 *
	Urban	100	21.70	15.78	(18.61–24.79)		Subs./priv.	42	18.29	14.43	(13.92–22.65)	
Unknown		2						2				
Sausages	Rural	130	26.34	21.77	(22.60–30.08)	0.011 *	Public	188	25.55	21.67	(22.46–28.65)	<0.001 *
	Urban	101	19.21	19.84	(15.34–23.078)		Subs./priv.	43	13.02	15.51	(8.39–17.66)	
Unknown		2						2				
Sugar	Rural	129	17.47	19.13	(14.17–20.77)	0.637	Public	186	17.63	18.96	(14.91–20.36)	0.247
	Urban	98	16.31	17.46	(12.85–19.76)		Subs./priv.	41	13.95	15.45	(9.22–18.68)	
Unknown		6						6				
Sugary soft drinks	Rural	130	29.80	22.35	(25.96–33.64)	0.236	Public	187	29.51	22.46	(26.29–32.73)	0.077
	Urban	99	26.26	22.31	(21.87–30.66)		Subs./priv.	42	22.76	21.28	(16.33–29.20)	
Unknown		4						4				
Commercial juices	Rural	128	29.92	21.31	(26.23–33.61)	0.179	Public	185	29.54	22.08	(26.35–32.72)	0.049 *
	Urban	97	25.86	23.77	(21.13–30.59)		Subs./priv.	40	21.85	23.31	(14.63–29.07)	
Unknown		8						5				
Sweets	Rural	129	26.16	19.51	(22.79–29.52)	0.460	Public	186	26.18	19.13	(23.43–28.93)	0.153
	Urban	97	24.23	19.22	(20.40–28.05)		Subs./priv.	40	21.35	20.20	(15.09–27.61)	
Unknown		9						7				
Fruits	Rural	130	9.80	13.32	(7.51–12.09)	0.242	Public	187	10.11	13.22	(8.21–12.00)	0.146
	Urban	99	11.98	14.71	(9.08–14.88)		Subs./priv.	42	13.57	16.72	(8.52–18.63)	
Unknown		4						4				
Vegetables	Rural	130	14.00	16.26	(11.20–16.80)	0.840	Public	188	13.87	16.26	(11.55–16.20)	0.912
	Urban	101	13.58	14.50	(10.76–16.41)		Subs./priv.	43	13.58	11.63	(10.11–17.06)	
Unknown		2						2				

n, sample size; SD, standard deviation; Student’s *t*-test *p*-values; * variable with significant effect *p* < 0.05.

**Table 6:** The proportion of students who ate breakfast and where they ate it.

	Breakfast			Place			
5–6 Years	Yes	No	*p*-Value (Chi-Square)	Home	School	Other	*p*-Value (Chi-Square)
Rural	79 (94%)	5 (6%)	0.434	74 (93.7%)	3 (3.8%)	1 (1.3%)	0.043 *
Urban	154 (95.1%)	6 (3.7%)		152 (99.3%)	0	1 (0.7%)	
Unknown	11			24			
Public	159 (95%)	7 (5%)	0.590	154 (96.3%)	3 (1.9%)	3 (1.9%)	0.428
Subs./priv.	74 (95%)	4 (5%)		72 (100%)	0	0	
Unknown	11			23			
12 years							
Rural	76 (90.5%)	8 (9.5%)	0.145	74 (97.4%)	1 (1.3%)	1 (1.3%)	0.826
Urban	132 (95.7%)	5 (3.6%)		130 (97%)	1 (0.7%)	3 (2.2%)	
Unknown	9			20			
Public	144 (94.7%)	7 (4.6%)	0.406	141 (97.2%)	1 (0.7%)	3 (2.1%)	0.816
Subs./priv.	64 (91.4%)	6 (8.6%)		63 (96.9%)	1 (1.5%)	1 (1.5%)	
Unknown	9			20			
15 years							
Rural	109 (83.8%)	19 (14.6%)	0.453	104 (96.3%)	3 (2.8%)	1 (0.9%)	0.147
Urban	86 (86%)	14 (14%)		79 (91.9%)	3 (2.3%)	5 (5.8%)	
Unknown	5			38			
Public	154 (82.4%)	31 (16.6%)	0.099	144 (94.1%)	4 (2.6%)	5 (3.3%)	0.961
Subs./priv.	41 (95.3%)	2 (4.7%)		39 (95.1%)	1 (2.4%)	1 (2.4%)	
Unknown	5			39			

* Variable with significant effect (*p* < 0.05).

**Table 7:** The proportion of students who ate lunch and where they ate it.

	Lunch			Place			
5–6 Years	Yes	No	*p*-Value (Chi-Square)	Home	School	Other	*p*-Value (Chi-Square)
Rural	73 (90.1%)	4 (4.9%)	0.533	73 (90.1%)	2 (2.5%)	6 (7.4%)	0.037 *
Urban	139 (90.3%)	4 (2.6%)		114 (77.6%)	16 (10.9%)	17 (11.6%)	
Unknown	35			27			
Public	148 (96.1%)	6 (3.9%)	0.383	134 (85.9%)	8 (5.1%)	14 (9%)	0.043 *
Subs./priv.	64 (96.9%)	2 (3.1%)		53 (73.6%)	10 (13.9%)	9 (12.5%)	
Unknown	35			27			
12 years							
Rural	69 (86.3%)	7 (8.8%)	0.010 *	62 (78.5%)	8 (10.1%)	9 (11.4%)	0.932
Urban	132 (97.1%)	2 (1.5%)		105 (78.4%)	12 (9%)	17 (12.7%)	
Unknown	20			17			
Public	138 (93.9%)	5 (3.4%)	0.710	119 (81.5%)	9 (6.2%)	18 (12.3%)	0.057
Subs./priv.	63 (91.3%)	4 (5.8%)		48 (71.6%)	11 (16.4%)	8 (11.9%)	
Unknown	20			17			
15 years							
Rural	114 (91.2%)	7 (5.6%)	0.201	88 (74.6%)	6 (5.1%)	24 (20.3%)	0.127
Urban	97 (97%)	2 (2%)		73 (75.3%)	11 (11.3%)	13 (13.4%)	
Unknown	13			18			
Public	173 (95.1%)	5 (2.7%)	0.142	126 (71.6%)	15 (8.5%)	35 (19.9%)	0.052
Subs./priv.	38 (88.4%)	4 (9.3%)		35 (89.7%)	2 (5.1%)	2 (5.1%)	
Unknown	13			18			

* Variable with significant effect (*p* < 0.05).

**Table 8:** The proportion of students who ate dinner and where they ate it according to geographic location and school type.

	Dinner			Place		
5–6 Years	Yes	No	*p*-Value (Chi-Square)	Home	Other	*p*-Value (Chi-Square)
Rural	74 (90.2%)	4 (4.9%)	0.566	72 (94.7%)	4 (5.3%)	0.086
Urban	149 (93.7%)	4 (2.5%)		146 (98.6%)	2 (1.4%)	
Unknown	24			31		
Public	152 (92.1%)	7 (4.2%)	0.430	148 (96.7%)	5 (3.3%)	0.423
Subs./priv.	71 (93.4%)	1 (1.3%)		70 (98.6%)	1 (1.4%)	
Unknown	24			31		
12 years						
Rural	79 (98.8%)	1 (1.3%)	0.392	79 (100%)	0	0.115
Urban	129 (95.6%)	4 (3%)		126 (96.9%)	4 (3.1%)	
Unknown	17			21		
Public	142 (96.6%)	4 (2.7%)	0.731	141 (91.3%)	1 (1.7%)	0.063
Subs./priv.	66 (97.1%)	1 (1.5%)		64 (95.5%)	3 (4.5%)	
Unknown	17			21		
15 years						
Rural	120 (93%)	9 (7%)	0.180	115 (98.3%)	2 (1.7%)	0.674
Urban	97 (97%)	3 (3%)		96 (99%)	1 (1%)	
Unknown	4			19		
Public	176 (94.6%)	10 (5.4%)	0.847	172 (98.9%)	2 (1.1%)	0.512
Subs./priv.	41 (95.3%)	2 (4.7%)		39 (97.5%)	1 (2.5%)	
Unknown	4			19		

**Table 9:** Effect of food consumption on the experience of caries in permanent teeth.

	OR	CI (95%)	*p*-Value
Sweets	1.757	1.07–2.98	0.035 *
Commercial juices	1.316	0.87–2.08	0.197
Pastries	1.095	0.69–1.73	0.699
Sugary soft drinks	1.039	0.67–1.62	0.867
Potato chips	1.374	0.79–2.38	0.256
Sausages	1.185	0.75–1.88	0.472
Sugar	0.890	0.61–1.30	0.545
Fruit	0.857	0.61–1.21	0.383
Vegetables	1.001	0.67–1.50	0.997

OR, odds ratio; CI, confidence interval; Student’s *t*-test; * variable with significant effect (*p* < 0.05).

**Table 10:** The proportion of schoolchildren with an experience of caries according to the meals consumed throughout the day and the location where they are eaten.

Experience of Caries	Yes	No	*p*-Value (Chi-Square)	Home	School	Other	*p*-Value (Chi-Square)
Breakfast							
No experience of caries	479 (93.6%)	33 (6.4%)	0.004 *	463 (96.9%)	6 (1.3%)	8 (1.3%)	0.558
Experience of caries	157 (86.7%)	24 (13.3%)		150 (94.9%)	4 (2.5%)	0	
Unknown	25			87			
Lunch							
No experience of caries	452 (96%)	19 (4%)	0.943	383 (79.5%)	46 (9.5%)	53 (11%)	0.009 *
Experience of caries	172 (96.1%)	7 (3.9%)		132 (75.9%)	9 (5.2%)	33 (19%)	
Unknown	68			62			
Dinner							
No experience of caries	480 (97.6%)	12 (2.4%)	0.004 *	472 (98.3%)	-	8 (1.7%)	0.292
Experience of caries	168 (92.8%)	13 (7.2%)		162 (97%)	-	5 (3%)	
Unknown	45			71			

* Variable with significant effect (*p* < 0.05).

## Data Availability

The datasets generated and/or analyzed during the current study are not publicly available, as they are being utilized for ongoing purposes; however, they are available from the corresponding author upon reasonable request.

## Abbreviations

The following abbreviations are used in this manuscript:

WHO	World Health Organization
FAO	Food and Agriculture Organization of the United Nations
EFSA	European Food Safety Authority
